# Systems genetics analysis defines importance of TMEM43/*LUMA* for cardiac- and metabolic-related pathways

**DOI:** 10.1152/physiolgenomics.00066.2021

**Published:** 2021-11-12

**Authors:** Qingqing Gu, Fuyi Xu, Buyan-Ochir Orgil, Zaza Khuchua, Undral Munkhsaikhan, Jason N. Johnson, Neely R. Alberson, Joseph F. Pierre, Dennis D. Black, Deli Dong, Jaclyn A. Brennan, Brianna M. Cathey, Igor R. Efimov, Jeffrey A. Towbin, Enkhsaikhan Purevjav, Lu Lu

**Affiliations:** ^1^Department of Genetics, Genomics and Informatics, University of Tennessee Health Science Center, Memphis, Tennessee; ^2^Department of Cardiology, The Affiliated Hospital of Nantong University, Nantong, China; ^3^Department of Pediatrics, University of Tennessee Health Science Center, Memphis, Tennessee; ^4^Children’s Foundation Research Institute, Le Bonheur Children’s Hospital, Memphis, Tennessee; ^5^The Heart Institute, Cincinnati Children’s Hospital Medical Center, Cincinnati, Ohio; ^6^Department of Biochemistry, Sechenov University, Moscow, Russia; ^7^Department of Pharmacology, College of Pharmacy, Harbin Medical University, Harbin, China; ^8^Department of Biomedical Engineering, The George Washington University, Washington, District of Columbia; ^9^Department of Pediatric Cardiology, St. Jude Children's Research Hospital, Memphis, Tennessee; ^10^School of Pharmacy, Binzhou Medical University, Yantai, Shandong, China

**Keywords:** BXD family, cardiomyopathy, gene network, systems genetics, TMEM43/LUMA

## Abstract

Broad cellular functions and diseases including muscular dystrophy, arrhythmogenic right ventricular cardiomyopathy (ARVC5) and cancer are associated with transmembrane protein43 (TMEM43/*LUMA*). The study aimed to investigate biological roles of *TMEM43* through genetic regulation, gene pathways and gene networks, candidate interacting genes, and up- or downstream regulators. Cardiac transcriptomes from 40 strains of recombinant inbred BXD mice and two parental strains representing murine genetic reference population (GRP) were applied for genetic correlation, functional enrichment, and coexpression network analysis using systems genetics approach. The results were validated in a newly created knock-in *Tmem43*-S358L mutation mouse model (Tmem43^S358L^) that displayed signs of cardiac dysfunction, resembling ARVC5 phenotype seen in humans. We found high *Tmem43* levels among BXDs with broad variability in expression. Expression of *Tmem43* highly negatively correlated with heart mass and heart rate among BXDs, whereas levels of *Tmem43* highly positively correlated with plasma high-density lipoproteins (HDL). Through finding differentially expressed genes (DEGs) between Tmem43^S358L^ mutant and wild-type (Tmem43^WT^) lines, 18 pathways (out of 42 found in BXDs GRP) that are involved in ARVC, hypertrophic cardiomyopathy, dilated cardiomyopathy, nonalcoholic fatty liver disease, Alzheimer’s disease, Parkinson’s disease, and Huntington’s disease were verified. We further constructed *Tmem43*-mediated gene network, in which *Ctnna1*, *Adcy6*, *Gnas*, *Ndufs6*, and *Uqcrc2* were significantly altered in Tmem43^S358L^ mice versus Tmem43^WT^ controls. Our study defined the importance of *Tmem43* for cardiac- and metabolism-related pathways, suggesting that cardiovascular disease-relevant risk factors may also increase risk of metabolic and neurodegenerative diseases via *TMEM43*-mediated pathways.

## INTRODUCTION

Transmembrane protein 43 (TMEM43/*LUMA*), a highly conserved integral membrane protein throughout all vertebrates, insects, unicellular eukaryotes, some plants, and several bacteria, is expressed in cellular inner nuclear membrane (INM) and endoplasmic reticulum (ER) (1, 2). TMEM43 is also found in adherens and composite junctions of cardiac myocytes and epithelial cells in humans and other mammals (3). Moreover, TMEM43 is found in many organs, including small intestine, thymus, prostate, and testis in humans (4), suggesting important cellular functions for TMEM43. Structurally, the protein consists of four transmembrane spanning domains, several phosphorylation sites, a transactivation domain, SUMO attachment site, and an O-glycosylation site that may play important biological functions (4). TMEM43 has been shown to interact with emerin, lamins A/C, and B1 (4), and SUN2 (Sad1 and UNC84 Domain Containing 2) protein (5); all are the members of LINC (linker of nucleoskeleton and cytoskeleton) complex that provides physical and biological bridging between nucleus and cytoskeleton (6). LINC functions as a mechanosensing unit that translates extracellular and intracellular mechanical forces into biochemical signals in the nuclei, regulating diverse cellular processes such as cytoskeletal organization, nuclear architecture, chromatin dynamics, and gene expression (7).

Arrhythmogenic right ventricular cardiomyopathy (ARVC), an inherited cardiac muscle disease characterized by malignant arrhythmias, is associated with p.R28W, p.E142K, p.R312W, p.S358L, p.V89M, and p.R299T mutations (8, 9). Particularly, the S358L mutation causes a fully penetrant ARVC5 with sudden cardiac death (10, 11). Mutations p.E85K and p.I91V in *TMEM43* have been reported in patients with Emery–Dreifuss muscular dystrophy type 7 (EDMD7) (5, 12). Hereditary serrated polyposis syndrome, a high-risk disorder of colorectal cancer, has shown significant segregation with rare *TMEM43* variants (13). Moreover, a substantial phenotype variability in humans and animals associated with *TMEM43* expression is shown in tumor malignancy via NF-κB activation (14), with innate immune signaling via stimulator of interferon genes (STING) pathway (15) as well as with cardiomyopathy through DNA damage and TP53 pathways due to TMEM43 haplo-insufficiency (16). Despite that, the biological roles of *Tmem43* in murine heart remain controversial (17, 18).

Many Mendelian diseases with the same mutation may result in severe disease, mild presentation, or be entirely asymptomatic (19). The cogent reason behind variable presentations is that a gene or disease-causing mutation does not entirely determine the phenotype or disease onset and progression. The final phenotype is characterized by complex genetic and epistatic interactions involving numerous genes and their encoded proteins combined with environmental factors. The power to detect those complex genetic and epistatic interactions is limited in human cohorts (due to ethical concerns) or in genetically modified animal models, because animal modeling is commonly created on a fixed genetic background (20). Thus, systems genetics approach linking complex genotypes to variable phenotypes, gene-gene, and gene-environment interactions at the genetic and epigenetic levels, pathogenic networks, and pathways are designed to identify biological processes and understand their regulation (21). Utilization of animal recombinant inbred (RI) genetic reference populations (GRP), particularly, offers a significant power to boost effective heritability, because each isogenic RI line and its stable genome can be replicated many times in a controlled environment.

In this study, we applied systems genetics analysis of BXD RI strains descended from cross between C57BL/6J (B6) and DBA/2J (D2) parental strains to determine biological roles of TMEM43 by identifying *Tmem43*-mediated pathways in murine GRP. Then, we validated those pathways by identifying differentially expressed genes (DEGs) between mutant knock-in S358L Tmem43 mice and WT littermate controls created on B6 background and constructed *Tmem43*-mediated genetic network (22). The identified *Tmem43-*correlated genes are predicted to perturb cardiac and metabolic related homeostasis and function pathways.

## MATERIALS AND METHODS

### Animals

Animal studies were approved by Institutional Animal Care and Use Committee (IACUC) at Cincinnati Children’s Hospital Medical Center (CCHMC) and University of Tennessee Health Science Center (UTHSC). All animals had free access to standard laboratory diet and water. The total of 40 BXD strains, their B6 and D2 parental strains at age of 6–7 mo and 3 strains of Tmem43-knock-in (KI) heterozygous (HET), homozygous (HOMO), and wild-type (WT) mice at age of 3–4 mo (*n* > 5/strain) were used in this study.

### Creation of Tmem43-S358L Knock-In Mouse

To generate a murine model of *TMEM43*-S358L mutation, we targeted exon 12 in the *Tmem43* gene and knocked-in the p.Ser358Leu substitution by replacing two nucleotides at c.1072 T → C and c.1073C→T into murine genome using a homologous recombination method (Supplemental Material; all Supplemental material is available at https://doi.org/10.6084/m9.figshare.14794596). Briefly, an MC1 retrieval vector was created with AB and YZ arms for homologous recombination corresponding to 548 and 526 bp sequences within intron 11 and 12, respectively, to target 13537 bp region (Supplemental Fig. S1*A*). Targeting vector comprised large and small homology arms (LHA and SHA, respectively), a removable neomycin resistance cassette (Neo) flanked with *frt* sites, and thymidine kinase negative selection cassette (TK). After NotI digestion to linearize the complete targeting construct, 40 µg of DNA was electroporated into 6 million Embryomax 129/SVEV embryonic stem cells (Millipore, Billerica, MA). Cells resistant to G418 were selected by culturing for 9 days in medium containing 0.26 mM G418 and 2 µM gancyclovir. Recombined embryonic stem cell clones were identified using PCR-based screening with primers, forward specific to Neo-cassette (5′- TGCCTGCTTGCCGAATATCATGGTGGAA-3′) and reverse specific to genomic region downstream to SHA (5′- CCTCGCTTAGTAGAAATGCTTCC-3′). Clones that were confirmed to be correct using direct sequencing were injected into C57BL/6 blastocysts at the Transgenic Mouse Core Facility of CCHMC (Cincinnati, OH). Mice were genotyped by PCR. All genotyping PCR was performed using LongAmp PCR mastermix (New England Biolabs, Ipswich, MA). Mouse colonies were maintained under barrier conditions with free access to standard laboratory diet and water. Chimeric animals were bread and backcrossed seven times with B6 mice and then the homozygote Tmem43^S358L^ mice were obtained from crossing the Tmem43^WT/S358L^ heterozygotes. The correct mRNA as well as insertion of two nucleotides was confirmed by direct sequencing of total cDNA and levels of *Tmem43* mRNA and TMEM43 protein expression in the heart were confirmed by quantitative real-time PCR (qRT-PCR) and Western blotting, respectively, using generic protocols.

### Cardiac Magnetic Resonance Imaging in Tmem43 Mice

Cardiac magnetic resonance imaging (cMRI) was used to evaluate heart function in 3-mo-old Tmem43^S358L^ and control Tmem43^WT^ littermate mice from heterozygous intercrosses (*n* = 5 animals per group). For all functional studies, mice were anesthetized by oxygenated 1.5% isoflurane and core temperatures were maintained using a heated platform set at 37°C. Cardiac magnetic resonance imaging (cMRI) was performed using a Bruker 7 T scanner. Image acquisition was prospectively ECG-gated using pediatric ECG probes attached to the paws. A bolus of gadopentetic acid (Gd-DTPA, 0.3–0.6 mmol/kg) was given intraperitoneally while the mouse was placed in the scanner bore. Delayed enhancement MRI was performed using a T1-weighted cine sequence. Cine imaging was performed in the short axis using a segmented fast low angle shot (FLASH) sequence. Slice thickness = 1.0 mm, matrix size = 256 × 256, in-plane resolution = 117 × 117 μm^2^, echo time/repetition time (TE/TR) = 3/5.2 ms, flip angle = 20°, segments = 1. Approximately 15–20 cine frames were acquired during the cardiac cycle. Tagged images were acquired in the middle, basal, and apical planes of the left ventricle. The left ventricular (LV) maximum circumferential strain (Ecc) was calculated using HARP software (Diagnosoft Plus, Diagnosoft Inc., CA). Atrial and ventricular end diastolic volumes and LV and right ventricular (RV) ejection fractions (LVEF, RVEF) were calculated using freely available software Segment (http://segment.heiberg.se). Diastolic function was quantified as the rate of change of Ecc in diastole, $\frac{\text{d(Ecc)}}{\text{d}t}$ using the mid ventricular tagged images. The tagged images were also used to quantify maximum apical twist, T, rate of change of T in diastole, $\frac{\text{d(T)}}{\text{d}t}$. The LV sphericity was defined as the ratio of LV maximum width (approximately at the midlevel) to the LV base-to-apex length at end diastole in the four-chamber view.

### Electrocardiography in Tmem43 Mice

Noninvasive in vivo ECG recordings were performed in conscious mice (*n* > 3/group/sex). Animals were placed in a restraining tunnel on top of four electrode pads (emka TECHNOLOGIES) and continuous signals were recorded for ∼10 min using the accompanying IOX2 software. Raw ECG signals were exported into MATLAB 2017a for analysis of heart rate. A 60 Hz notch filter was applied, and heart rate variability and Poincare plots were generated to illustrate beat-to-beat variation.

### Mouse Blood and Tissue Harvest

The mice were euthanized under isoflurane anesthesia after an overnight fast. Plasma and heart tissue were collected from mice per protocols published previously (23). Briefly, blood obtained from cardiac puncture was collected into EDTA tubes (10 mM final concentration) and the plasma was separated by centrifugation (1,500 *g*, 15 min) and stored in −80°C until further use. The hearts were removed and perfused with cardioplegic solution for histology or alternatively snap-frozen in liquid nitrogen for genetic molecular studies. Small intestine (jejunum and ileum) was removed from the gastrointestinal tract and gently flushed with iced saline. Tissue samples were snap frozen in liquid nitrogen and stored at −80°C until use. Primers used for qRT-PCR are shown in Supplemental Table S1. For histological evaluation, perfused mouse hearts were fixed in 10% formalin. After 48 h in formalin, hearts were embedded in paraffin, then sectioned and applied to H&E staining.

### Lipid-Level Assays in Plasma and Feces of Mice

Plasma lipids levels were measured using cholesterol, HDL, and LDL assay kit (Abcam, Cambridge, UK) according to the provided protocols (*n* > 5 mice/group). Fecal samples were collected from 3-mo-old Tmem43 mice fed with standard laboratory chow (*n* = 10 mice per genotype). Briefly, 1 g of fecal pellets was collected and powered using a mortar and pestle. Total lipids were extracted via evaporation using saline and chloroform (Thermo Fisher Scientific, Waltham, MA) and the lipid mass (mg) per 1 g of feces (mg/g) was obtained.

### Extraction of Total RNA from Mouse Myocardium and Intestine and Microarray Analysis

In this study, we used transcriptome data set of cardiac tissue across 40 strains of BXD and two parental C57BL/6J (B) and DBA/2J (D2) strains that we generated previously and deposited as “EPFL/LISP BXD CD Heart Affy Mouse Gene 2.0 ST (Jan14) RMA” in GeneNetwork (www.genenetwork.org) (24). GeneNetwork is a web resource of multi-omic data sets for BXD GRP, including genome, transcriptome, proteome, metabolome, and phenome data. Total RNA was extracted from the ventricular myocardium of BXDs, B6 and D2 parental strains, and Tmem43^WT^ and Tmem43^S358L^ animals euthanized under isoflurane anesthesia after an overnight fast using QIAGEN RNA extraction kits (https://www.qiagen.com) as per the manufacturer’s instructions. To reduce the inhomogeneous nature of tissues due to the presence of different segments of the heart, the individual RNA sample from five mice at same strain were pooled evenly (by µg of RNA) into a single RNA sample and then purified using RNEasy kit. The RNA integrity number (RIN) for all samples were evaluated using Agilent 2100 Bioanalyzer. The RNA integrity value between 1.8 and 2 as well as the RIN values greater than 8 were considered for passing as quality control. The Affymetrix Mouse Gene 2.0 ST arrays were used to generate the gene expression data. The transcriptome quantification of ventricular myocardium including RNA extraction, array platform, data normalization, and validation was performed in adult Tmem43^WT^ and Tmem43^S358L^ mice as aforementioned. Five animals per group were used. Comparisons between groups were conducted with the Limma package (25). Differential expression analysis between groups was examined by *t* test, and genes with *P* values<0.05 were defined as differentially expressed genes (DEGs). Candidate cardiac DEGs and genes involved in intestinal lipid absorption were validated by qRT-PCR using a Quant Studio 6 Flex (ThermoFisher) and Eva-Green master mix (Bio-Rad). All samples were assayed in triplicate at least two times, and the average value was used for quantification. In all experiments, *Gapdh* or β actin was used as housekeeping genes. The data were analyzed using the comparative ΔCt method (ΔΔCt method) and results are expressed in fold changes related to that in Tmem43^WT^ mice.

### Microarray Data Set Analysis

Raw microarray data was normalized using the Robust Multichip Array (RMA) method. The expression data were then renormalized using a modified Z-score described previously (26). We calculated the log base 2 of normalized values above, computed *Z*-scores for each array, multiplied the *Z*-scores by 2, and added an offset of 8 units to each value. The reason for this transformation was to produce a set of *Z*-like scores for each array that have a mean of 8 and standard deviation of 2. The advantage of this modified *Z*-score is that a twofold difference in expression corresponds approximately to a 1-unit change.

### Genetic Correlation Analysis

We computed genetic correlations between expression of *Tmem43* and expression of all other probe sets across the genome using heart gene expression data sets of BXD mice. All genetic correlations were computed through the Pearson’s product-moment correlation using Genenetwork tools (27). The Pearson’s product correlations value *P* < 0.05 was used for indicating significant correlations. Literature correlation examines the *r* value for genes that are described by similar terminology in published papers (28). Genes with literature correlation value *r* > 0.1 were selected for further analysis.

### Gene Set Enrichment Analysis and Gene Network Construction

Gene set enrichment analysis for KEGG (Kyoto Encyclopedia of Genes and Genomes) pathway was done with ShinyGO (http://bioinformatics.sdstate.edu/go/) (29). The *P* values generated from the hypergeometric test were automatically adjusted to account for multiple comparisons using Benjamini and Hochberg correction (30). The categories with false discovery rate (FDR) < 0.1 indicated that the set of submitted genes are significantly overrepresented in those categories. The genes that were significantly enriched in cardiomyopathy-related pathways were used for gene network analysis. Spring Model Layout Network Graphs were constructed using online Genenetwork tools. Each node in a graph represents an individual transcript and interconnecting lines illustrate ranges of Pearson’s correlation coefficient values. Other public search engines such as PubMed, OMIM, DAVID, GeneCard, BioGrid, and Cytoscape were used for function evaluation of members in the *Tmem43* network.

### Western Blot Analysis

For protein expression quantification, Western blotting analysis was performed. Whole heart tissue was homogenized in T-PER reagent (Thermo Fisher Scientific) with a protease inhibitor mixture (Roche Applied Science). Total proteins were quantified using Pierce BCA Protein Assay Kit (Thermo Fisher Scientific) and 25 µg of total protein was applied on Nupage 4%–12% Bis-Tris gel (Invitrogen). Blotting on PVDF membrane was done for 2 h at 30 V while cooled on ice. After 2 h of blocking in Tris-buffered saline (0.1% Tween-20 and 5% nonfat dry milk), membranes were incubated with primary TMEM43 antibody (Abcam) overnight at 4°C on a rocking platform. The membranes were subsequently incubated with secondary antibodies for an hour, and proteins were detected using Li-Cor Odyssey. Levels were quantified in relative optical density (OD) units using Image Studio Lite software. GAPDH was used as a reference.

### Statistical Analysis

All results, except gene expression data, are presented as means ± SD and statistical analysis was achieved with Student’s *t* test or ANOVA using GraphPad Prism6 (GraphPad Software, La Jolla, CA). A *P* value < 0.05 was considered significant after Mann–Whitney tests with appropriate Bonferroni correction.

## RESULTS

### TMEM43 Is Highly Expressed in the Heart of BXD Mice

**Figure 1.**
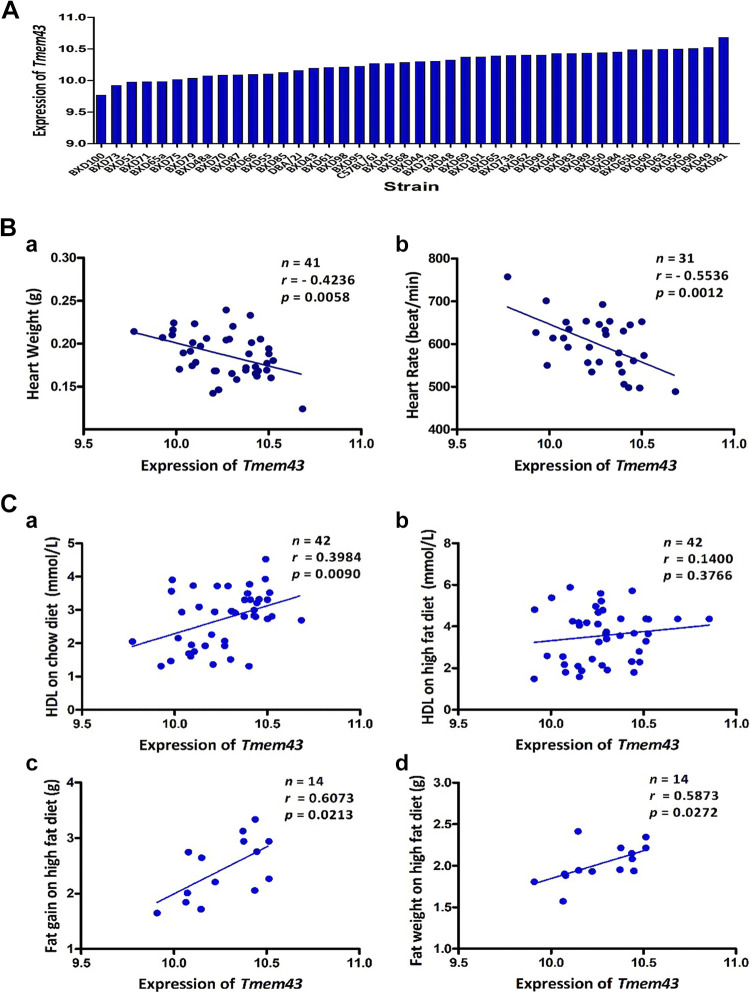
Systems genetics analysis of *Tmem43*. *A*: expression values of *Tmem43* in the heart across 40 BXDs and their parental strains. The *x*-axis denotes the strain. The *y*-axis denotes the mean expression of *Tmem43* given in a log2 scale. *B*: significant negative correlation (*P* < 0.001) between *Tmem43* expression in the heart (*x*-axis) and an average of heart weight (*a*) and heart rate (*b*, beats/min) in BXD mice represented on the *y*-axis. *C*: scatter plots of correlations between *Tmem43* expression in the heart (*x*-axis) to plasma HDL levels (*y*-axis) in BXDs on chow diet (*a*) or high-fat diet (*b*). Correlations between *Tmem43* expression levels and fat gain (*c*) or fat weight (*d*, *y*-axis) in BXDs fed with high-fat diet.

To determine cardiac *Tmem43* expression in murine GRP of BXD strains, we analyzed microarray data set of ventricular myocardium deposited in the Genenetwork, a web-based multi-omic resource of GRP, including genome, transcriptome, proteome, metabolome, and phenome data (24), and found the range of *Tmem43* expression showed the highest in BXD81 (10.68) and the lowest in BXD100 (9.77), a difference of >1.88-fold (Fig. 1*A*), presenting a broad variability among BXDs. The average *Tmem43* expression was 10.27 ± 0.03 (log_2_ scale, means ± SE), which is approximately eight times higher compared with average expression of all other genes, suggesting important roles for *Tmem43* in heart function and *Tmem43* expression is affected by genetic background.

### Cardiac *Tmem43* Expression Is Highly Negatively Correlated with Heart Weight and Heart Rate in BXD Strains

To determine association of *Tmem43* with cardiac phenotypes, we first performed an unbiased correlation between levels of *Tmem43* in the heart and cardiac traits in BXDs that we collected in previous study and archived in our Genenetwork database (www.genenetwork.org). Considerable strain differences in heart weight (HW) and heart rate (HR) have been reported among BXDs (31). This phenomenon is associated with genetic background of individual lines as the BXDs are maintained in controlled laboratory environment. We found the levels of cardiac *Tmem43* highly negatively (*P* < 0.01) correlated to HW and HR in BXDs (Fig. 1*B*), indicating that higher cardiac *Tmem43* expression was observed in BXD strains with lower HW or slower HR, suggesting important roles for *Tmem43* in heart morphology and function.

### Cardiac *Tmem43* Expression Is Highly Correlated with Metabolic-Related Phenotypes in BXD Strains

Further, we found *Tmem43* expression in the heart highly significantly correlated with the levels of high-density lipoproteins (HDL, Fig. 1*Ca*) in plasma of BXD mice fed with normal chow diet indicating that higher cardiac *Tmem43* expression was observed in BXD strains with higher HDL levels in plasma. Next, we correlated *Tmem43* with phenotypes of BXDs fed with high-fat diet (HFD). Although the significant correlation between *Tmem43* and plasma HDL was lost on HFD (Fig. 1*Cb*), we found highly significant correlation between cardiac *Tmem43* and fat gain (Fig. 1*Cc*, *P* < 0.05, phenotype ID 15000) as well as fat weight (Fig. 1*Cd*, *P* < 0.05, phenotype ID 14835) in BXD mice fed a HFD. These results suggested that *Tmem43* may also play important roles in fat absorption, metabolism, and storage, especially under environmental modifiers such as diet.

### Enrichment Analysis Reveals Involvement of *Tmem43* in the Development of Cardiac Diseases

**Figure 2.**
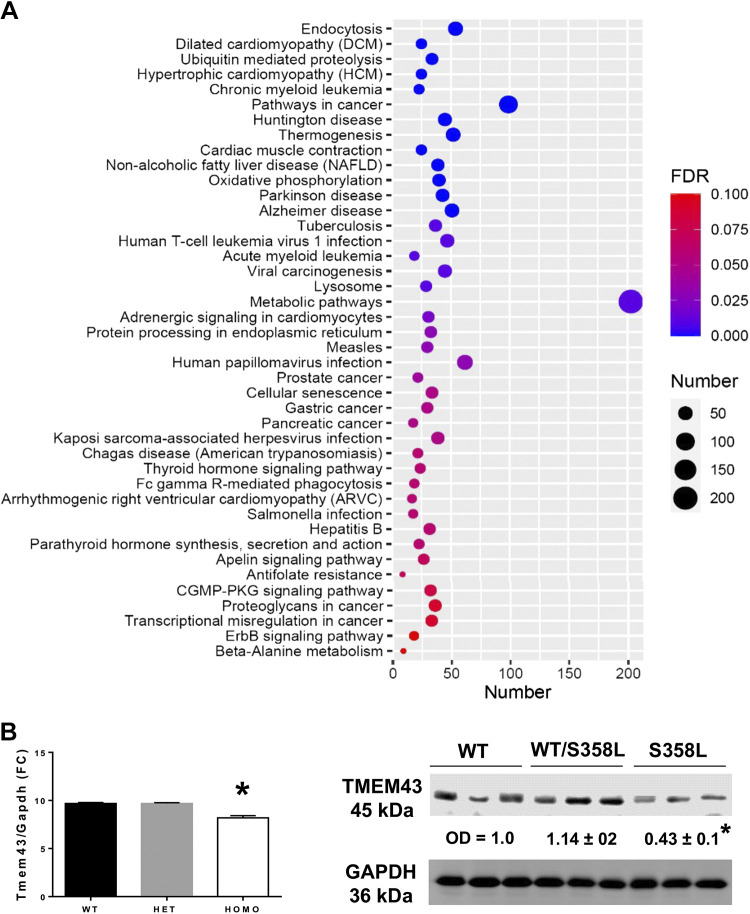
Enrichment analysis and expression of Tmem43 in Tmem43-KI mice. *A*: bubble charts of the KEGG pathways enriched for *Tmem43* covariates (*P* ≤ 0.05). Gene overrepresentation analysis for KEGG pathway was performed using ShinyGO analysis. The *x*-axis represents gene numbers and *y*-axis represents enriched terms. The size of the dots represents gene numbers. The FDR value is indicated by colors. *B*: levels of Tmem43 RNA and protein in Tmem43-KI mouse hearts. *Left*: results of qRT-PCR performed in Tmem43^WT^ (black), Tmem43^WT/S358L^ (gray), and Tmem43^S358L^ mice. Expression of *Tmem43* is shown in fold change. *Gapdh* was used as a reference. *Right*: images of Western blotting of TMEM43 and GAPDH used as a loading reference. Expression levels are indicated in optical density (OD) relative to that of in Tmem43^WT^ controls. *P < 0.05, significant difference from Tmem43^WT^ and Tmem43^WT/S358L^ groups. FDR, false discovery rate; KI, knock-in.

We further hypothesized that *Tmem43* interacts with other candidate genes involved in pathogenesis of cardiac diseases and we could find those genes by identifying the genetic correlates of *Tmem43* and enriching with similar biological functions, pathways, and phenotypes. Thus, we performed genetic correlation analysis and identified ∼1,600 transcripts/probe sets whose expression levels were significantly (average expression > 7, *P* < 0.05, *r* > 0.1) correlated with *Tmem43* expression and uploaded those transcripts to the ShinyGO website (http://bioinformatics.sdstate.edu/go/) for gene function enrichment analysis. We then identified 42 significantly enriched pathways (FDR < 0.1) that were mainly enriched in cardiac-related pathways such as hypertrophic cardiomyopathy (HCM, 25 genes, FDR = 9.03E-04), dilated cardiomyopathy (DCM, 25 genes, FDR = 1.39E-03), and ARVC (17 genes, FDR = 5.93E-02). Other significant pathways included metabolic pathways (MP, 203 genes, FDR = 5.43E-03), nonalcoholic fatty liver disease (NAFLD, 39 genes, FDR = 2.28E-04), oxidative phosphorylation (40 genes, FDR = 3.15E-06), protein processing in endoplasmic reticulum (33 genes, FDR = 3.47E-02), neurodegenerative, and cancer-related pathways (Fig. 2*A*).

### Analysis of Cardiac Phenotype and *Tmem43* Expression in Tmem43-KI Mice

A heterozygous *TMEM43*-S358L mutation causes a lethal autosomal dominant ARVC5 in humans (10). To validate results obtained in BXD strains, we created a mutant knock-in Tmem43^S358L^ mouse model by conventional genetic targeting and crossed to B6 mice for genetic background concurrence with BXDs. All lines of Tmem43-KI mice were born and developed normally. To confirm expression of *Tmem43* mRNA free of insertions such as the Neo-cassette and *frt* sites used for targeted knock-in, we performed direct sequencing of RT-PCR product of total RNA isolated from the heart of Tmem43-KI mice and the presence of two nucleotide knocked-in substitutions TCC > CTC, resulting in Ser to Leu substitution has been confirmed (Supplemental Figs. S1 and S2**)**. Quantitative RT-PCR analysis of the heart demonstrated significantly reduced levels of mutant *Tmem43* expression in the heart, which we also validated on a protein level by Western blot analysis (Fig. 2*B*).

**Figure 3.**
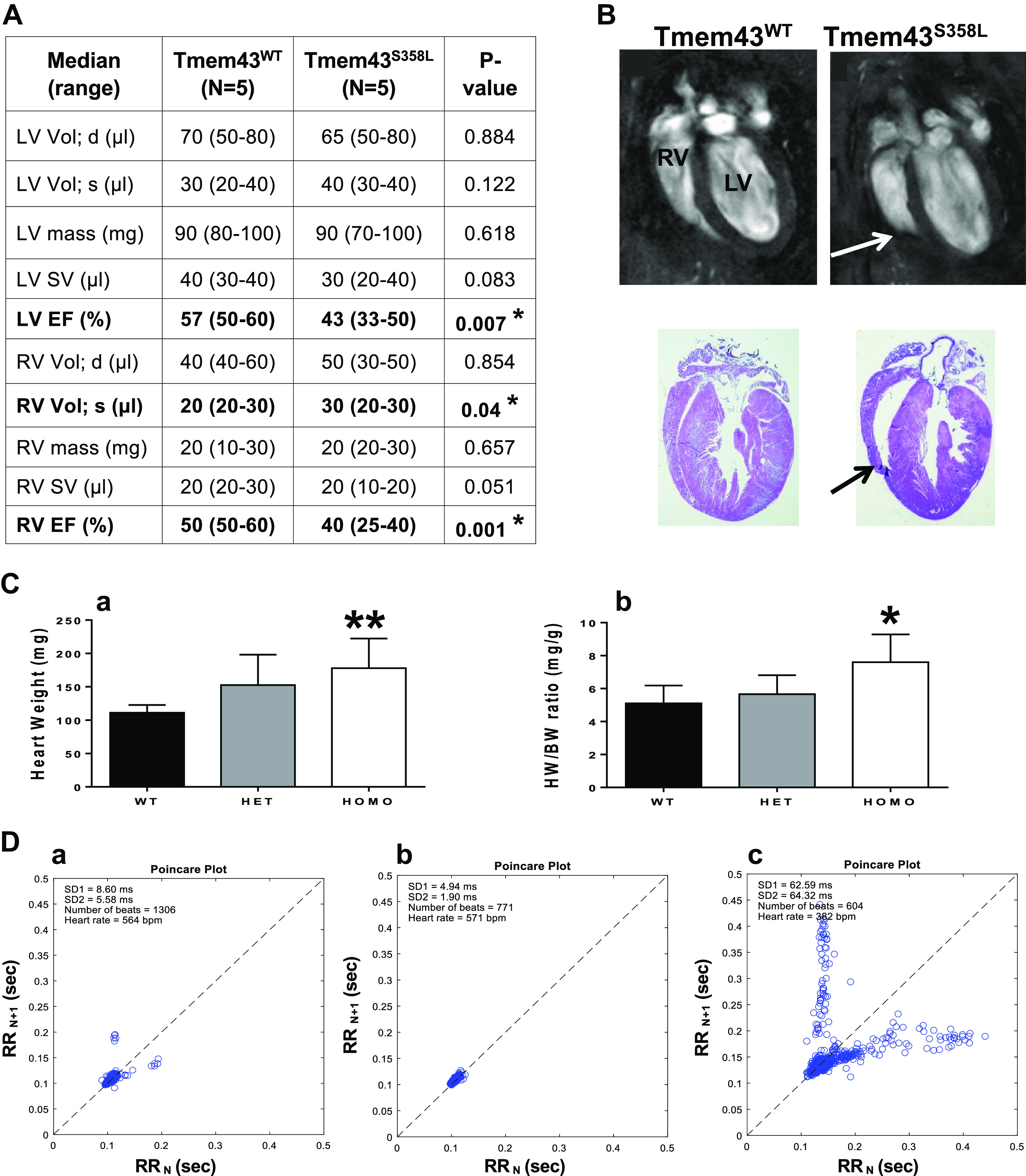
Cardiac phenotypes of Tmem43-KI mice. *A*: results of cardiac MRI presented in median ranges. *P < 0.05, significant difference from WT littermates. *B*: representative images of longitudinal view of mouse hearts on cardiac MRI (*top*) and histology (*bottom*) demonstrate aneurism of the RV wall (arrow) and RV dilation in Tmem43^S358L^ mutants compared with WT littermate mice. *C*: heart weight (HW) and heart and body weight (HW/BW) ratio in Tmem43-KI mice. A significant increase in HW (*a*, **P < 0.001) and HW/BW ratio (*b*, *P < 0.05) found in Tmem43^S358L^ (white columns) compared with Tmem43^WT^ (black columns) and Tmem43^WT/S358L^ (gray columns) mice. *D*: representative Poincaré scatterplots from ECG recording of conscious 3-mo-old WT female (*a*), Tmem43^S358L^ female (*b*), and Tmem43^S358L^ male (*c*) mice (*n* = 3). No association between *Tmem43* expression and heart rate was found in Tmem43-KI mice. d, diastole; EF, ejection fraction; KI, knock-in; LV, left ventricular; RV, right ventricular; s, systole; SV, stroke volume; WT, wild type; Vol, volume; RR, interval between two heart beats; SD1, short-term standard deviation; SD2, long-term standard deviation.

Further, cardiac function was evaluated in 3-mo-old mice by cMRI, which revealed significant decrease in LVEF and RVEF, demonstrating biventricular systolic dysfunction in Tmem43^S358L^ mutant strains compared with Tmem43^WT^ controls (Fig. 3*A*). Volume of RV was significantly (*P* < 0.04) increased in Tmem43^S358L^ compared with that in Tmem43^WT^ littermate mice, suggestive for RV dilation. We also observed a thinning of the RV walls in Tmem43^S358L^ mice and related aneurysm on cMRI and histology that are often reported in ARVC patients as the “triangle of dysplasia” (32) (Fig. 3*B*, arrows). Taken together, these results suggested a direct causal effect of *TMEM43*-S358L mutation resulting in biventricular cardiomyopathy phenotype.

We next validated correlations between *Tmem43* expression and HW and HR in Tmem43-KI mouse hearts using microarray. Similar to qRT-PCR analysis, cardiac microarray revealed significantly downregulated *Tmem43* in Tmem43^S358L^ mice compared with that of Tmem43^WT^ controls. In contrast, HW and ratio of HW to body weight (HW/BW) were significantly increased in Tmem43^S358L^ mice, corroborating similar negative trends between *Tmem43* expression and HW seen in BXDs (Fig. 3*C*).

To assess heart rate, ECG tracings of 3-mo-old conscious mice were applied. ECG tracing revealed similar HR in all groups, and no association between *Tmem43* and HR is observed (Fig. 3*D*). Of note, HR asymmetry was observed in Tmem43^S358L^ male mice only [with standard deviation of RR (SDRR) = 25.41, *n* = 2, Fig. 3D*c*], whereas no heart rate variability was found in female Tmem43^S358L^ (SDRR = 3.48, *n* = 4, Fig. 3D*a*) or Tmem43^WT^ (SDRR = 3.67, *n* = 3, Fig. 3D*b*) mice.

### Correlation between Cardiac *Tmem43* Expression and Plasma Lipid Levels in Tmem43-KI Mice

**Figure 4.**
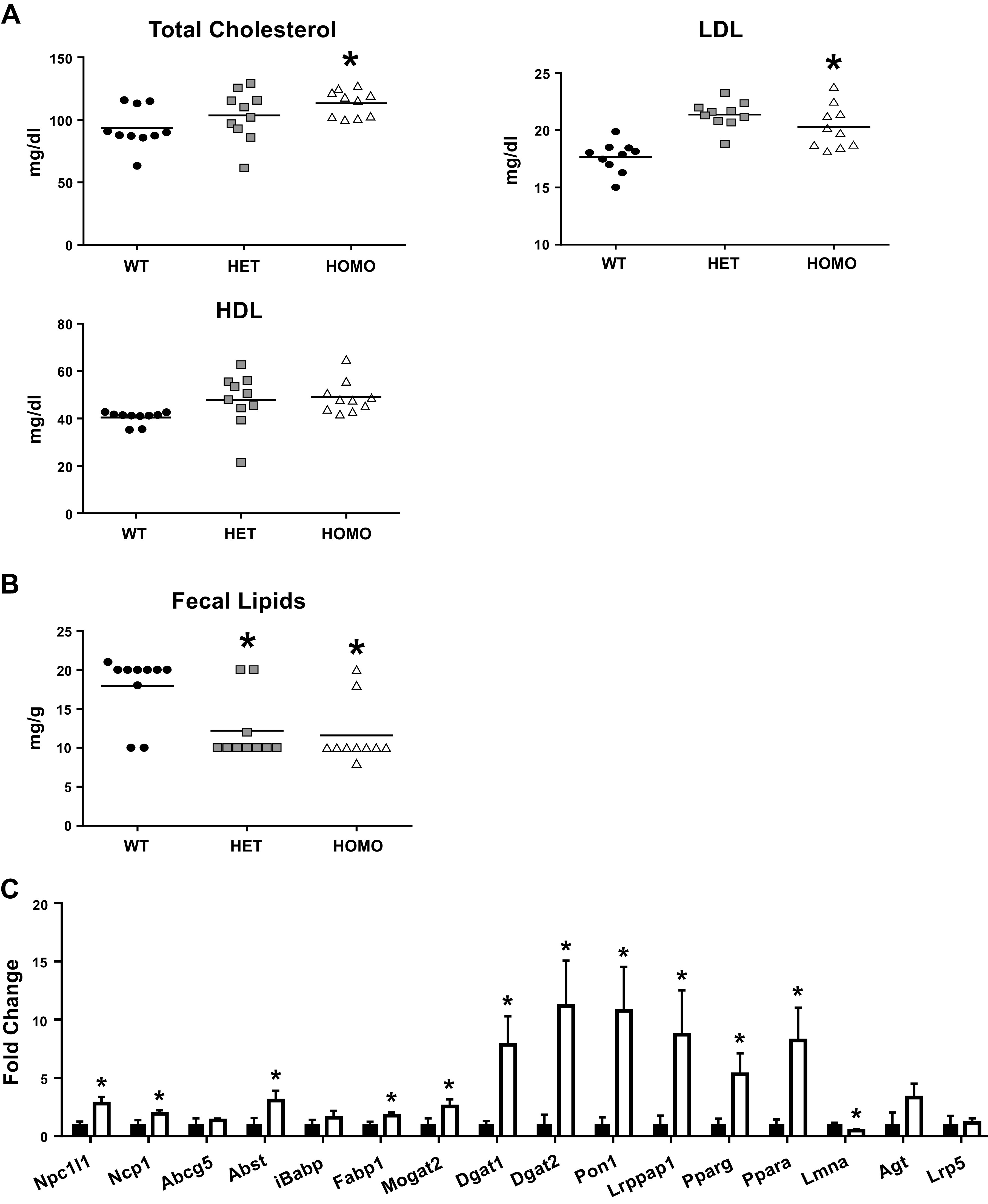
Plasma and fecal lipids and expression of lipid absorption-related genes in the intestine of Tmem43-KI mice. *A*: levels of plasma cholesterol, HDL, and LDL in plasma and fecal lipids (*n* = 10) of Tmem43-KI mice.*P < 0.05, significant difference compared with WT mice. *B*: results of qRT-PCR indicate significant increase of genes involved in lipid absorption in intestine of Tmem43^S358L^ mice (white columns) compared with that in Tmem43^WT^ (black columns) controls, *n* = 5 mice per group. *C*: expression of genes is shown in fold change. *Gapdh* was used as a reference. *P < 0.05, significant difference compared with WT mice. KI, knock-in; WT, wild type.

As significant correlation between *Tmem43* expression and HDL was found in BXDs, we next measured levels of plasma lipids in Tmem43 mice (Fig. 4*A*) using ELISA, which revealed significantly (*P* < 0.05) higher total cholesterol and LDL levels in mutants compared with Tmem43^WT^ controls. HDL was also increased in mutants as well, but it did not reach a statistical significance compared with Tmem43^WT^ groups.

Elevated HDL and LDL levels in plasma have been previously reported in parental D2 mice, and this phenomenon was associated with increased expression of genes controlling cholesterol absorption (33). Thus, we next examined fecal lipid content to expose whether increased plasma lipids in Tmem43 mutants was associated with increased lipids absorption. Compared with Tmem43^WT^ (*n* = 10), feces from mutants contained 35% less lipid (Fig. 4*B*; *P* < 0.05), suggesting that the latter animals more efficiently absorbed lipids and fatty acids (FA) from the gut lumen, providing the first evidence of *Tmem43-*S358L association with a decrease in fecal lipid excretion and reciprocal hyperlipidemia. Subsequently, we examined expression levels of genes involved in absorption and reesterification of lipids in the intestine of Tmem43 mice using qRT-PCR (Fig. 4*C*). Expression of *Npc1*, *Npc1l1*, *Abcg5* (cholesterol transporters), *Asbt*, *Fabp1* (bile acid and FA transporters), *Mogat2*, *Dgat1*, *Dgat2* (triglyceride reesterification), *Lrpap1* (inhibiting LDL degradation), and *Ppara* and *Pparg* (transcription factors that regulate lipid metabolism) was significantly increased in the gut mucosa of Tmem43^S358L^ compared with Tmem43^WT^ littermates. These results suggested enhanced fat and macronutrient uptake from the gut in Tmem43^S358L^ mice and likely exposing similar origin of hyperlipidemia-reported parental D2 mouse (33).

### Validation of *Tmem43* Gene Pathways

To further validate the Tmem43 pathways that were identified using systems genetics in BXD GRP, we conducted microarray analysis of the myocardium to compare expression differences between Tmem43^WT^ and Tmem43^S358L^ strains. We identified ∼3,200 differentially expressed transcript/probe sets (*P* < 0.05) in the LV between Tmem43^WT^ and Tmem43^S358L^ mice, and 3,000 in the RV and 3,400 in the LV and RV combined. After excluding unannotated probe sets, a total of ∼4,200 cardiac DEGs were identified between Tmem43^WT^ and Tmem43^S358L^ strains (Supplemental Table S2). Further, KEGG pathway enrichment analysis found 48 enriched terms (FDR < 0.1), 18 of those were overlapped with the KEGG pathways enriched for the *Tmem43* covariates found in BXDs, including cardiomyopathy-, metabolic- and neurodegenerative-related pathways (Table 1), suggesting that those pathways could be directly disturbed by change in *Tmem43* expression or induced by the S358L mutation.

**Table 1.** List of the overlapped KEGG pathways, Tmem43 covariates in BXDs, and DEGs in Tmem43-KI mice

KEGG Pathway	Covariates		DEGs		No. of Overlapped Genes	Overlapped Genes
	FDR	No. of Genes	FDR	No. of Genes		
Acute myeloid leukemia	0.01	19	0.10	22	7	*RUNX1 CCND1 STAT5B ZBTB16 MAPK3 DUSP6 RPS6KB1*
Alzheimer’s disease (AD)	3.19E-07	51	1.00E-03	59	20	*ATP5C1 ATP5F1 ATP5H CALM1 COX7A2 CYCS FADD MT-ATP6 NDUFS1 ATP5G3 BACE1 MAPK3* ***NDUFS6*** *NDUFA3 NDUFB9 PSENEN NDUFB3* ***UQCRC2*** *PSEN1 RTN3*
Apelin signaling	0.07	27	0.09	39	10	*ADCY6 CALM1 CCND1 GNB4 HDAC5 KLF2 PLAT PPARGC1A MAPK3 RPS6KB1*
ARVC	0.06	17	0.07	24	4	***CTNNA1*** *ITGA2 SGCG CACNG6*
Cellular senescence	0.05	34	0.10	47	11	*E2F4 CALM1 CCND1 CDC25A MDM2 PTEN TGFBR2 VDAC3 MAPK3 CHEK2 GM7030*
Dilated cardiomyopathy (DCM)	1.39E-03	25	0.10	27	7	***ADCY6*** ***GNAS*** *ITGA2 TTN SGCG CACNG6 TPM3*
Human papillomavirus infection	0.03	62	0.10	87	21	*ATP6V1B2 ATP6V0D1 ATP6V1E1 ATP6V1G1 CCND1 DLG1 FADD GNAS MAGI1 ITGA2 JAK1 LFNG MDM2 EIF2AK2 PSEN1 PTEN CREB5 MAPK3 PPP2R3C GM7030 RPS6KB1*
Human T-cell leukemia virus 1 infection	0.01	47	0.02	68	16	*ADCY6 CANX CCND1 DLG1 KAT2A JAK1 JAK3 ANAPC1 PTEN STAT5B TGFBR2 VDAC3 CREB5 MAPK3 CHEK2 GM7030*
Huntington’s disease (HD)	4.42E-04	45	0.01	60	19	*PPIF ATP5C1 ATP5F1 COX7A2 CYCS MT-ATP6 PPARGC1A REST VDAC3 NDUFS1 ATP5G3 CREB5* ***NDUFS6*** *NDUFA3 NDUFB9 POLR2E NDUFB3* ***UQCRC2*** *ATP5H*
Hypertrophic cardiomyopathy (HCM)	9.03E-04	25	0.10	26	5	*ITGA2 TTN SGCG CACNG6 TPM3*
Kaposi sarcoma-associated herpesvirus infection	0.05	39	0.02	60	11	*CALM1 CCND1 CCR5 CYCS FADD GNB4 HIF1A JAK1 EIF2AK2 MAPK3 GM7030*
Nonalcoholic fatty liver disease (NAFLD)	2.28E-04	39	0.07	43	9	*COX7A2 CYCS ITCH NDUFS1* ***NDUFS6*** *NDUFA3 NDUFB9 NDUFB3* ***UQCRC2***
Oxidative phosphorylation	3.15E-06	40	0.03	41	16	*ATP5C1 ATP5F1 ATP6V1B2 ATP6V0D1 ATP6V1E1 COX7A2 MT-ATP6 NDUFS1 ATP5G3 NDUFS6 NDUFA3 NDUFB9 ATP6V1G1 NDUFB3 UQCRC2 ATP5H*
Parkinson’s disease (PD)	1.20E-06	43	0.05	42	15	*ATP5C1 ATP5F1 COX7A2 CYCS MT-ATP6 VDAC3 NDUFS1 ATP5G3* ***NDUFS6****, ATP5H NDUFA3 NDUFB9 NDUFB3* ***UQCRC2*** *PPIF*
Protein processing in endoplasmic reticulum	0.03	33	0.07	46	7	*CANX EIF2AK2 SEC61G SEL1L CKAP4 MAN1C1 UBQLN2*
Prostate cancer	0.04	22	0.07	30	6	*CCND1 MDM2 PLAT PTEN CREB5 MAPK3*
Thermogenesis	3.63E-04	52	1.05E-03	73	19	*ADCY6 ATP5C1 ATP5F1 COX7A2 GNAS MT-ATP6 PPARGC1A RPS6KA2 NDUFS1 ATP5H ATP5G3 COX18 CREB5 NDUFS6 NDUFA3 NDUFB9 NDUFB3 UQCRC2 RPS6KB1*

Significantly altered genes between Tmem43^S358L^ mutant and Tmem43^WT^ control mice are highlighted in bold and underlined. ARVC, arrhythmogenic right ventricular cardiomyopathy; DEGs, differentially expressed genes; FDR, false discovery rate; KI, knock-in.

We further identified five genes (*Cacng6*, *Itga2*, *Ttn*, Sgcg, and *Tpm3)* that are also involved in HCM-related pathways, seven genes (*Adcy6, Gnas, Ttn*, *Cacng6*, *Itga2*, *Sgcg*, and *Tpm3*) in DCM-related pathways, and four genes (*Cacng6*, *Ctnna1*, *Itga2*, and *Sgcg*) in ARVC-related pathways identified in BXD strains had a significant expression change between Tmem43^WT^ and Tmem43^S358L^ strains. In addition, we also found 16 overlapping genes between BXDs and Tmem43-KI mice involved in oxidative phosphorylation and 9 genes in NAFLD (Table 1). Collectively, the results of comparative microarray analyses demonstrated that *Tmem43*-S358L mutation may disturb all these putative *Tmem43*-mediated pathways through *Tmem43*-correlated genes.

### Construction and Validation of *Tmem43*-Mediated Gene Coexpression Network in the Myocardium of Tmem43-KI Mice

**Figure 5.**
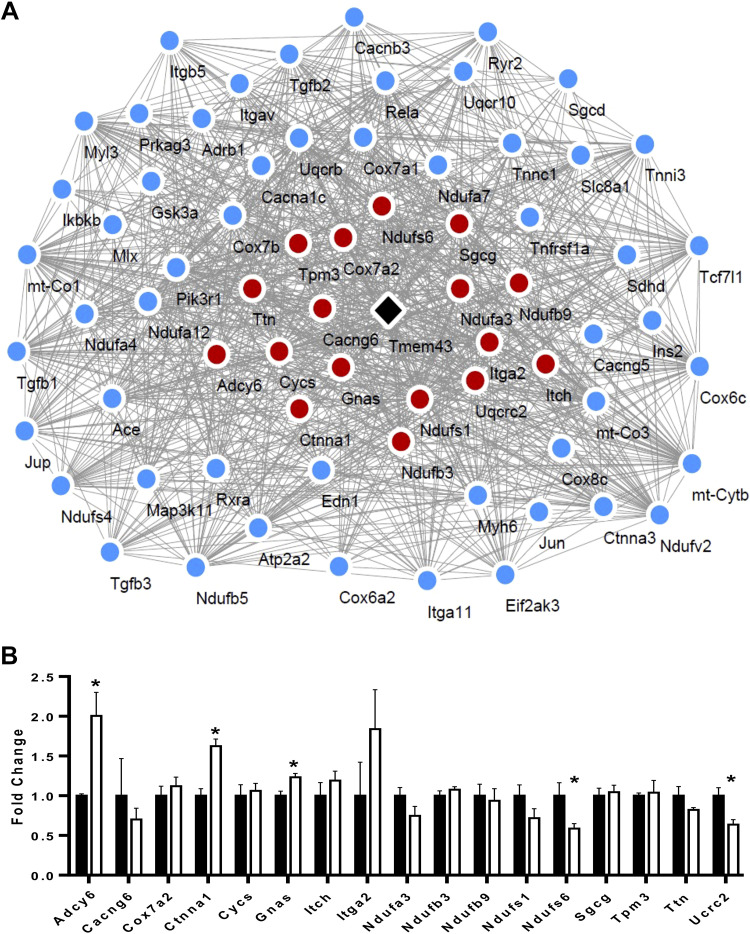
*Tmem43*-mediated gene coexpression network and expression of genes involved in this network. *A*: *Tmem43*-mediated gene coexpression network. The nodes in the network represent genes and edges represent genetic correlations (*P* < 0.05), respectively. Genes in this network are significantly correlated (*P* < 0.05) with *Tmem43* in the heart of BXD mice and involved in KEGG pathways of HCM, DCM, ARVC, or NAFLD pathways. Red nodes represent genes differentially expressed in the heart between mutant Tmem43^S358L^ and control Tmem43^WT^ mice. *B*: results of qRT-PCR for genes differentially expressed between Tmem43^WT^ and mutant Tmem43^S358L^ myocardium. Black columns indicate Tmem43^WT^ group and white columns indicate Tmem43^S358L^ mutants (*n* = 4). *Gapdh* was used as a reference. *P < 0.05, significant difference in fold change compared with Tmem43^WT^ mice. ARVC, arrhythmogenic right ventricular cardiomyopathy; DCM, dilated cardiomyopathy; HCM, hypertrophic cardiomyopathy; NAFLD, nonalcoholic fatty liver disease.

As we found a significant correlation between Tmem43 with cardiomyopathy- and metabolic-related traits in BXD strains and Tmem43 mutants, we performed genetic correlation analysis for the genes that have significant genetic correction with *Tmem43* in HCM, DCM, ARVC, NAFLD, and metabolic pathway (MP) and constructed a *Tmem43*-mediated gene network that is involved in the development of cardiomyopathy and metabolic diseases. We included 70 genes including *Tmem43* itself in this analysis and identified 990 gene pairs out of the 2,415 pairwise comparisons that had a significant Pearson’s correlation (*P* < 0.05). Out of 70 genes analyzed, we found 17 DEGs between Tmem43^WT^ and Tmem43^S358L^ strains (Fig. 5*A*, *red nodes*), suggesting that *Adcy6*, *Cacng6*, *Cox7a2*, *Ctnna1*, *Cycs*, *Gnas*, *Itch*, *Itga2*, *Ndufa3*, *Ndufb3*, *Ndufb9*, *Ndufs1*, *Ndufs6*, *Sgcg*, *Tpm3*, *Ttn*, and *Uqcrc2* may be the interacting genes with *Tmem43* according to the putative interactions identified.

We next validated the expression levels of all 17 DEGs identified in the myocardium of Tmem43 mice using qRT-PCR (Fig. 5*B*). The results demonstrated a significant (*P* < 0.05) upregulation of *Adcy6*, *Ctnna1*, and *Gnas*, whereas *Ndufs6* and *Uqcrc2* were significantly decreased in the heart of Tmem43^S358L^ mice compared with Tmem43^WT^ littermates, corroborating the involvement of cardiac- and metabolic-related pathways in the phenotypes seen in Tmem43^S358L^ mice and partly exposing the underlying mechanisms of deleterious ARVC5 in humans.

## DISCUSSION

Mutations in *TMEM43* are associated with varied diseases in humans including ARVC5, a severe form of cardiomyopathy with malignant arrhythmias and sudden cardiac death (5, 10–12). In particular, the *TMEM43*-S358L mutation causes a fully penetrant lethal ARVC5 in humans (10). Several mouse models carrying the *Tmem43* S358L mutation were reported including Tmem43-KI mouse of SV129 background (34), humanized transgenic mice overexpressing human mutation (35), and KI mouse created by CRISPR/Cas9 approach (18). In this study, we used a high-power and high-precision systems genetics approach to explore *Tmem43* gene function and identified novel TMEM43-induced mechanisms, pathways, and networks relevant to heart function and cardiovascular diseases. For systems genetics studies, we utilized murine GRP consisting of 40 BXD RI lines and their parental B6 and D2 strains for genotype-phenotype association and compared the results with newly created knock-in Tmem43^S358L^ mouse on B6 background using conventional targeting. Tmem43^S358L^ mutants recapitulated some of human traits including biventricular systolic dysfunction, RV dilation, and thinning of the RV walls with aneurysm revealed by cMRI.

Among BXD strains maintained in laboratory standard conditions with minimal environmental influences and fed with chow diet, *Tmem43* was highly expressed in the heart with a broad expression variability between BXD lines, suggesting that *Tmem43* levels are affected by a genetic background. In contrast, levels of cardiac TMEM43 expression were significantly reduced in Tmem43^S358L^ mutants compared with Tmem43^WT^ controls, suggesting a dominant negative effect of mutation on TMEM43 expression abundance. The same phenomenon observed in patients with ARVC5 and S358L mutation has been accompanied with decrease in plakoglobin, plakophilin-2, connexin-43, and emerin, demonstrating shared “final common pathway” with desmosome-associated ARVC (36, 37). Interestingly, haplo-insufficiency of Tmem43 has been shown to activate the DNA damage response pathway leading to senescence-associated cardiomyopathy in vivo (16). To find genes that could share the genetic regulator and have similar biologic function with *Tmem43*, we performed genetic correlation and functional enrichment analysis in BXDs and identified 42 *Tmem43*-mediated pathways, 18 of those (42.8%) including cardiomyopathy-related (HCM, DCM, and ARVC), and metabolic-related (NAFLD) pathways have been confirmed through analysis of DEGs between Tmem43^S358L^ and Tmem43^WT^ mice.

Further, cardiac *Tmem43* expression significantly negatively associated with heart weight and heart rate as well as HDL levels in plasma of BXD mice, suggesting important roles for *Tmem43* not only in murine heart morphology and function but also in lipid metabolism. Moreover, cardiac *Tmem43* highly significantly correlated with fat gain and fat weight in BXDs fed HFD. Intriguingly, Tmem43^S358L^ mutant mice also had hyperlipidemia due to increased lipids absorption from the gut lumen, emphasizing the importance of assessing levels of plasma lipids in patients with ARVC5 under environmental modifier such as HFD. NAFLD is the most common liver disease worldwide with the prevalence of 25%–30% among general adult population in Western countries and of 70%–75% among patients with type 2 diabetes mellitus (38), resulting from increased fat absorption from the gut similar to that we found in our Tmem43^S358L^ mutants. There is a strong association between NAFLD and increased risk in death due to cardiac arrhythmias, cardiomyopathy, and coronary disease often seen in patients with NAFLD (39). Therefore, we used the genes that showed a significant genetic correction with *Tmem43* in HCM, DCM, ARVC, and NAFLD pathways among BXDs and DEGs between Tmem43^S358L^ and Tmem43^WT^ mice to construct the *Tmem43*-mediated gene network shown in Fig. 5. Using qRT-PCR, we confirmed significant alterations of *Ctnna1*, *Adcy6*, *Gnas*, *Ndufs6*, and *Uqcrc2* between Tmem43^S358L^ and Tmem43^WT^ hearts.

Almost 50% of ARVC cases are associated with mutation(s) in genes encoding desmosomal and cell-adhesion proteins required for mechanoelectrical coupling in the heart (37). *Tmem43*-mediated genes identified by systems genetics approach in this study are rather uncommon. Encoded by *CTNNA1*, catenin-a1 (also known as aE-catenin) is highly expressed in adherens junctions of cardiomyocytes, functioning as a mediator of actin filaments anchorage to the cadherin-catenin-vinculin complex at the sarcolemma as well as to the desmosomes via its interactions with plakophilin-2 (40). Inducible cardiac-specific αE-catenin conditional knockout mice developed cardiomyopathy with tendency to ventricular free-wall rupture due to intercalated disk defects (41). The *ADCY6* (also known as *AC6*) encodes adenylyl cyclase 6, a member of ubiquitously expressed family of enzymes that catalyze cyclic AMP (cAMP) from ATP, and regulates numerous cellular functions including cell growth, differentiation, apoptosis, and transcriptional regulation through β adrenoceptor/G-protein signal transduction (42). The G-protein α-subunit (G_s_α) that couples the G-protein receptors to adenylyl cyclase is a gene product of *GNAS* (43). Although g.2714G > T variant in *ADCY6* is associated with increased cardiac output, heart rate, and blood pressure at baseline conditions as well as in response to exercise (44), the common *GNAS* single nucleotide polymorphisms (SNPs), c.2273C > T and c.393C > T, are reported as predictors of ventricular tachyarrhythmias and sudden cardiac death (45).

Mitochondria are the site of fatty acid oxidation, a mechanism that protects cells and tissues against fatty acid accumulation, whereas reactive oxidative species (ROS) are freed during oxidation of FAs (46). Genes *Ndufs6* and *Uqcrc2* are involved in mitochondrial oxidation significantly downregulated in Tmem43^S358L^ mutant heart. Deficiency in mitochondrial complex I subunit NDUFS6 causes an excessive accumulation of ROS, underlying many cardiac, metabolic, and neurodegenerative disorders in humans (47). Wisloff et al. (48) reported markedly reduced UQCRC2 (ubiquinol-cytochrome c oxidoreductase core 2 subunit) protein levels in low-capacity runner rats compared with the high-capacity runners. Patients with ARVC5 respond to high-level physical activity with more than ninefold increase of malignant ventricular arrhythmias (11). Our results provide a genetic link between decreased mitochondrial oxidation and exercise-induced malignant arrhythmias, and thus, future mitochondrial functional studies in Tmem43-KI mice may aid to understanding the underlying mechanisms in ARVC of both *TMEM43* and non-*TMEM43* origins. In addition to cardiomyopathy- and metabolic-related pathways, we noted that *Tmem43*-associated mitochondrial genes, *NDUFS6* and *UQCRC2*, are enriched in neurodegenerative disease-related pathways, including Alzheimer’s disease (AD), Parkinson’s disease (PD), and Huntington’s disease (HD; Table 1). AD and its precursor mild cognitive impairment are progressive neurodegenerative disorders, which accounts for ∼70% of all dementia cases (49). Similar to patients with AD, typical plaque-like amyloid aggregations are found in the hearts of patients with DCM carrying *PSEN1* (Asp333Gly) and *PSEN2* (Ser130Leu) mutations (50). Moreover, mitochondrial complex I activity defects are consistently observed in the substantia nigra and prefrontal cortex of patients with PD and dementia (51), suggesting that similar *Tmem43*-mediated biochemical and genetic triggers may exist for these two vital organs.

In conclusion, this is the first systems genetics analysis using murine GRPs of BXD strains and genetically modified Tmem43-KI mice to clarify biological roles of *Tmem43* in murine heart. Analysis of transcriptome data sets of both BXDs and Tmem43-KI strains demonstrated that *Tmem43* plays important roles not only in the heart but it is also an important gene involved in metabolic and mitochondrial oxidation processes, suggesting that same risk factors relevant to cardiovascular diseases may also increase person’s risk of developing metabolic and neurodegenerative diseases via TMEM43-mediated pathways. Although the study was not directed to validating human phenotypes, underlying molecular mechanisms, direct gene-gene, and gene-protein interactions in Tmem43^S358L^ mice, our systems genetics approach by comparing with murine genetic reference population of RI strains determined the candidate genes that potentially interact with *Tmem43*, signifying the need for future investigations on cardiometabolic effects of *Tmem43*.

### Study Limitations

We note limitations of this study. First, cardiac histological and ultrastructural phenotypes and mechanisms of how the Tmem43^S358L^ mice develop biventricular phenotypes were not studied in depth. Second, ECG phenotypes in mutant Tmem43^S358L^ mice do not fully recapitulate phenotypes (e.g., arrhythmias or sudden death) seen in patients with ARVC5. ARVC5 is a disease expressed often in young adults that progresses with aging and exercise. Thus, further studies in aging mice as well as under exercise are needed to fully recapitulate human phenotypes and our ongoing studies focused on all these aspects in Tmem43-KI mice are in progress.

## DATA AVAILABILITY

The RNA-Seq data that support this study are available as follows: gene expression data sets of *Tmem43* in BXD strains and computer code produced in this study are available at: https://genenetwork.org/show_trait?trait_id=17460918&dataset=EPFL-LISPBXDHeCD0114.

## SUPPLEMENTAL DATA

10.6084/m9.figshare.14794596 Supplemental Figs. S1 and S2 and Supplemental Tables S1 and S2: https://doi.org/10.6084/m9.figshare.14794596.

## GRANTS

This work was supported in part by National Institutes of Health R01 Grants: HL128350 (to L.L.), HL151438 (to E.P., J.A.T., and L.L.), and CORNET award between UTHSC and Harbin Medical University, China (to D.D., E.P., and L.L.).

## DISCLOSURES

No conflicts of interest, financial or otherwise, are declared by the authors.

## AUTHOR CONTRIBUTIONS

E.P. and L.L. conceived and designed research; Q.G., F.X., B.-O.O., Z.K., U.M., J.N.J., N.R.A., J.F.P., J.A.B., B.M.C., and I.R.E. performed experiments; Q.G., F.X., B.-O.O., J.N.J., J.F.P., J.A.B., B.M.C., I.R.E., E.P., and L.L. analyzed data; F.X., D.D.B., I.R.E., E.P., and L.L. interpreted results of experiments; Q.G., F.X., B.-O.O., Z.K., and E.P. prepared figures; F.X. drafted manuscript; Z.K., N.R.A., J.F.P., D.D.B., D.D., J.A.T., E.P., and L.L. edited and revised manuscript; E.P. and L.L. approved final version of manuscript.

## References

[1] Dreger M, Bengtsson L, Schöneberg T, Otto H, Hucho F. Nuclear envelope proteomics: novel integral membrane proteins of the inner nuclear membrane. Proc Natl Acad Sci USA 98: 11943–11948, 2001. doi:10.1073/pnas.211201898. 11593002 PMC59747

[2] Schirmer EC, Florens L, Guan T, Yates JR 3rd, Gerace L. Nuclear membrane proteins with potential disease links found by subtractive proteomics. Science 301: 1380–1382, 2003. doi:10.1126/science.1088176. 12958361

[3] Franke WW, Dörflinger Y, Kuhn C, Zimbelmann R, Winter-Simanowski S, Frey N, Heid H. Protein LUMA is a cytoplasmic plaque constituent of various epithelial adherens junctions and composite junctions of myocardial intercalated disks: a unifying finding for cell biology and cardiology. Cell Tissue Res 357: 159–172, 2014. doi:10.1007/s00441-014-1865-1. 24770932

[4] Bengtsson L, Otto H. LUMA interacts with emerin and influences its distribution at the inner nuclear membrane. J Cell Sci 121: 536–548, 2008. doi:10.1242/jcs.019281. 18230648

[5] Liang WC, Mitsuhashi H, Keduka E, Nonaka I, Noguchi S, Nishino I, Hayashi YK. TMEM43 mutations in Emery-Dreifuss muscular dystrophy-related myopathy. Ann Neurol 69: 1005–1013, 2011. doi:10.1002/ana.22338. 21391237

[6] Stroud MJ, Banerjee I, Veevers J, Chen J. Linker of nucleoskeleton and cytoskeleton complex proteins in cardiac structure, function, and disease. Circ Res 114: 538–548, 2014. doi:10.1161/CIRCRESAHA.114.301236. 24481844 PMC4006372

[7] Lombardi ML, Lammerding J. Keeping the LINC: the importance of nucleocytoskeletal coupling in intracellular force transmission and cellular function. Biochem Soc Trans 39: 1729–1734, 2011. doi:10.1042/BST20110686. 22103516 PMC4589539

[8] Marcus FI, McKenna WJ, Sherrill D, Basso C, Bauce B, Bluemke DA, Calkins H, Corrado D, Cox MG, Daubert JP, Fontaine G, Gear K, Hauer R, Nava A, Picard MH, Protonotarios N, Saffitz JE, Sanborn DM, Steinberg JS, Tandri H, Thiene G, Towbin JA, Tsatsopoulou A, Wichter T, Zareba W. Diagnosis of arrhythmogenic right ventricular cardiomyopathy/dysplasia: proposed modification of the task force criteria. Circulation 121: 1533–1541, 2010. doi:10.1161/CIRCULATIONAHA.108.840827. 20172911 PMC2860804

[9] Honda T, Kanai Y, Ohno S, Ando H, Honda M, Niwano S, Ishii M. Fetal arrhythmogenic right ventricular cardiomyopathy with double mutations in TMEM43. Pediatr Int 58: 409–411, 2016. doi:10.1111/ped.12832. 26840987

[10] Merner ND, Hodgkinson KA, Haywood AF, Connors S, French VM, Drenckhahn JD, Kupprion C, Ramadanova K, Thierfelder L, McKenna W, Gallagher B, Morris-Larkin L, Bassett AS, Parfrey PS, Young TL. Arrhythmogenic right ventricular cardiomyopathy type 5 is a fully penetrant, lethal arrhythmic disorder caused by a missense mutation in the TMEM43 gene. Am J Hum Genet 82: 809–821, 2008. doi:10.1016/j.ajhg.2008.01.010. 18313022 PMC2427209

[11] Paulin FL, Hodgkinson KA, MacLaughlan S, Stuckless SN, Templeton C, Shah S, Bremner H, Roberts JD, Young TL, Parfrey PS, Connors SP. Exercise and arrhythmic risk in TMEM43 p.S358L arrhythmogenic right ventricular cardiomyopathy. Heart Rhythm 17: 1159–1166, 2020. doi:10.1016/j.hrthm.2020.02.028. 32120009

[12] Mukai T, Mori-Yoshimura M, Nishikawa A, Hokkoku K, Sonoo M, Nishino I, Takahashi Y. Emery-Dreifuss muscular dystrophy-related myopathy with TMEM43 mutations. Muscle Nerve 59: E5–E7, 2019. doi:10.1002/mus.26355. 30311943

[13] Toma C, Díaz-Gay M, Soares de Lima Y, Arnau-Collell C, Franch-Expósito S, Muñoz J, Overs B, Bonjoch L, Carballal S, Ocaña T, Cuatrecasas M, Díaz de Bustamante A, Castells A, Bujanda L, Cubiella J, Balaguer F, Rodríguez-Alcalde D, Fullerton JM, Castellví-Bel S. Identification of a novel candidate gene for serrated polyposis syndrome germline predisposition by performing linkage analysis combined with whole-exome sequencing. Clin Transl Gastroenterol 10: e00100, 2019. doi:10.14309/ctg.0000000000000100. 31663907 PMC6919450

[14] Jiang C, Zhu Y, Zhou Z, Gumin J, Bengtsson L, Wu W, Songyang Z, Lang FF, Lin X. TMEM43/LUMA is a key signaling component mediating EGFR-induced NF-κB activation and tumor progression. Oncogene 36: 2813–2823, 2017. doi:10.1038/onc.2016.430. 27991920

[15] Fenech EJ, Lari F, Charles PD, Fischer R, Laétitia-Thézénas M, Bagola K, Paton AW, Paton JC, Gyrd-Hansen M, Kessler BM, Christianson JC. Interaction mapping of endoplasmic reticulum ubiquitin ligases identifies modulators of innate immune signalling. eLife 9: e57306, 2020. doi:10.7554/eLife.57306. 32614325 PMC7332293

[16] Rouhi L, Cheedipudi SM, Chen SN, Fan S, Lombardi R, Chen X, Coarfa C, Robertson MJ, Gurha P, Marian AJ. Haploinsufficiency of Tmem43 in cardiac myocytes activates the DNA damage response pathway leading to a late-onset senescence-associated pro-fibrotic cardiomyopathy. Cardiovasc Res 117: 2377–2394, 2021. doi:10.1093/cvr/cvaa300. 33070193 PMC8861264

[17] Liu Y, Chen VHS, Shou W. LUMA in cardiac development and function. Cardiovasc Res 114: 347–348, 2018. doi:10.1093/cvr/cvx247. 29281017 PMC6018943

[18] Stroud MJ, Fang X, Zhang J, Guimarães-Camboa N, Veevers J, Dalton ND, Gu Y, Bradford WH, Peterson KL, Evans SM, Gerace L, Chen J. Luma is not essential for murine cardiac development and function. Cardiovasc Res 114: 378–388, 2018. doi:10.1093/cvr/cvx205. 29040414 PMC6019056

[19] Cerrone M, Remme CA, Tadros R, Bezzina CR, Delmar M. Beyond the one gene-one disease paradigm: complex genetics and pleiotropy in inheritable cardiac disorders. Circulation 140: 595–610, 2019. doi:10.1161/CIRCULATIONAHA.118.035954. 31403841 PMC6697136

[20] Al-Barghouthi BM, Farber CR. Dissecting the genetics of osteoporosis using systems approaches. Trends Genet 35: 55–67, 2019. doi:10.1016/j.tig.2018.10.004. 30470485 PMC6309493

[21] Parnell LD, Casas-Agustench P, Iyer LK, Ordovas JM. How gene networks can uncover novel CVD players. Curr Cardiovasc Risk Rep 8: 372, 2014. doi:10.1007/s12170-013-0372-3. 24683432 PMC3966201

[22] Génin E, Feingold J, Clerget-Darpoux F. Identifying modifier genes of monogenic disease: strategies and difficulties. Hum Genet 124: 357–368, 2008. doi:10.1007/s00439-008-0560-2. 18784943 PMC2911473

[23] Huby AC, Mendsaikhan U, Takagi K, Martherus R, Wansapura J, Gong N, Osinska H, James JF, Kramer K, Saito K, Robbins J, Khuchua Z, Towbin JA, Purevjav E. Disturbance in Z-disk mechanosensitive proteins induced by a persistent mutant myopalladin causes familial restrictive cardiomyopathy. J Am Coll Cardiol 64: 2765–2776, 2014. doi:10.1016/j.jacc.2014.09.071. 25541130 PMC4279060

[24] Gu Q, Mendsaikhan U, Khuchua Z, Jones BC, Lu L, Towbin JA, Xu B, Purevjav E. Dissection of Z-disc myopalladin gene network involved in the development of restrictive cardiomyopathy using system genetics approach. World J Cardiol 9: 320–331, 2017. doi:10.4330/wjc.v9.i4.320. 28515850 PMC5411966

[25] Smyth GK. Limma: linear models for microarray data. In: Bioinformatics and Computational Biology Solutions Using R and Bioconductor edited by Gentleman R, Carey VJ, Huber W, Irizarry RA, Dudoit S. New York, NY: Springer, 2005, p. 397–420.

[26] Lu H, Lu L, Williams RW, Jablonski MM. Iris transillumination defect and its gene modulators do not correlate with intraocular pressure in the BXD family of mice. Mol Vis 22: 224–233, 2016. 27011731 PMC4783577

[27] Mulligan MK, Mozhui K, Prins P, Williams RW. GeneNetwork: a toolbox for systems genetics. Methods Mol Biol 1488: 75–120, 2017. doi:10.1007/978-1-4939-6427-7_4. 27933521 PMC7495243

[28] Homayouni R, Heinrich K, Wei L, Berry MW. Gene clustering by latent semantic indexing of MEDLINE abstracts. Bioinformatics 21: 104–115, 2005. doi:10.1093/bioinformatics/bth464. 15308538

[29] Ge SX, Jung D, Yao R. ShinyGO: a graphical gene-set enrichment tool for animals and plants. Bioinformatics 36: 2628–2629, 2020. doi:10.1093/bioinformatics/btz931. 31882993 PMC7178415

[30] Benjamini Y, Drai D, Elmer G, Kafkafi N, Golani I. Controlling the false discovery rate in behavior genetics research. Behav Brain Res 125: 279–284, 2001. doi:10.1016/s0166-4328(01)00297-2. 11682119

[31] Zhao W, Zhao T, Chen Y, Zhao F, Gu Q, Williams RW, Bhattacharya SK, Lu L, Murine SYA. Hypertrophic cardiomyopathy model: the DBA/2J strain. PLoS One 10: e0133132, 2015. doi:10.1371/journal.pone.0133132. 26241864 PMC4524617

[32] van der Wall EE, Kayser HW, Bootsma MM, de Roos A, Schalij MJ. Arrhythmogenic right ventricular dysplasia: MRI findings. Herz 25: 356–364, 2000. doi:10.1007/s000590050028. 10948772

[33] Kirk EA, Moe GL, Caldwell MT, Lernmark JA, Wilson DL, LeBoeuf RC. Hyper- and hypo-responsiveness to dietary fat and cholesterol among inbred mice: searching for level and variability genes. J Lipid Res 36: 1522–1532, 1995. 7595076

[34] Zheng G, Jiang C, Li Y, Yang D, Ma Y, Zhang B, Li X, Zhang P, Hu X, Zhao X, Du J, Lin X. TMEM43-S358L mutation enhances NF-κB-TGFβ signal cascade in arrhythmogenic right ventricular dysplasia/cardiomyopathy. Protein Cell 10: 104–119, 2019. doi:10.1007/s13238-018-0563-2. 29980933 PMC6340891

[35] Padrón-Barthe L, Villalba-Orero M, Gómez-Salinero JM, Domínguez F, Román M, Larrasa-Alonso J, Ortiz-Sánchez P, Martínez F, López-Olañeta M, Bonzón-Kulichenko E, Vazquez J, Martí-Gómez C, Santiago DJ, Prados B, Giovinazzo G, Gómez-Gaviro MV, Priori S, Garcia-Pavia P, Lara-Pezzi E. Severe cardiac dysfunction and death caused by arrhythmogenic right ventricular cardiomyopathy type 5 are improved by inhibition of glycogen synthase kinase-3β. Circulation 140: 1188–1204, 2019. doi:10.1161/CIRCULATIONAHA.119.040366. 31567019 PMC6784777

[36] Christensen AH, Andersen CB, Tybjaerg-Hansen A, Haunso S, Svendsen JH. Mutation analysis and evaluation of the cardiac localization of TMEM43 in arrhythmogenic right ventricular cardiomyopathy. Clin Genet 80: 256–264, 2011. doi:10.1111/j.1399-0004.2011.01623.x. 21214875

[37] Towbin JA. Inherited cardiomyopathies. Circ J 78: 2347–2356, 2014. doi:10.1253/circj.cj-14-0893. 25186923 PMC4467885

[38] Non-alcoholic Fatty Liver Disease Study Group; Lonardo A, Bellentani S, Argo CK, Ballestri S, Byrne CD, Caldwell SH, Cortez-Pinto H, Grieco A, Machado MV, Miele L, Targher G. Epidemiological modifiers of non-alcoholic fatty liver disease: focus on high-risk groups. Dig Liver Dis 47: 997–1006, 2015. doi:10.1016/j.dld.2015.08.004. 26454786

[39] Mantovani A. Nonalcoholic fatty liver disease (NAFLD) and risk of cardiac arrhythmias: a new aspect of the liver-heart axis. J Clin Transl Hepatol 5: 134–141, 2017. doi:10.14218/JCTH.2017.00005. 28660151 PMC5472934

[40] Jamora C, Fuchs E. Intercellular adhesion, signalling and the cytoskeleton. Nat Cell Biol 4: E101–E108, 2002. doi:10.1038/ncb0402-e101. 11944044

[41] Sheikh F, Chen Y, Chen Y, Liang X, Hirschy A, Stenbit AE, Gu Y, Dalton ND, Yajima T, Lu Y, Knowlton KU, Peterson KL, Perriard J-C, Chen J. alpha-E-catenin inactivation disrupts the cardiomyocyte adherens junction, resulting in cardiomyopathy and susceptibility to wall rupture. Circulation 114: 1046–1055, 2006 [Erratum in *Circulation* 114: e650, 2006]. doi:10.1161/CIRCULATIONAHA.106.634469. 16923756

[42] Patel TB, Du Z, Pierre S, Cartin L, Scholich K. Molecular biological approaches to unravel adenylyl cyclase signaling and function. Gene 269: 13–25, 2001. doi:10.1016/s0378-1119(01)00448-6. 11376933

[43] Weinstein LS, Liu J, Sakamoto A, Xie T, Chen M. Minireview: GNAS: normal and abnormal functions. Endocrinology 145: 5459–5464, 2004. doi:10.1210/en.2004-0865. 15331575

[44] Hodges GJ, Gros R, Hegele RA, Van Uum S, Shoemaker JK, Feldman RD. Increased blood pressure and hyperdynamic cardiovascular responses in carriers of a common hyperfunctional variant of adenylyl cyclase 6. J Pharmacol Exp Ther 335: 451–457, 2010. doi:10.1124/jpet.110.172700. 20732959

[45] Wieneke H, Svendsen JH, Lande J, Spencker S, Martinez JG, Strohmer B, Toivonen L, Le Marec H, Garcia-Fernandez FJ, Corrado D, Huertas-Vazquez A, Uy-Evanado A, Rusinaru C, Reinier K, Foldesi C, Hulak W, Chugh SS, Siffert W. Polymorphisms in the GNAS gene as predictors of ventricular tachyarrhythmias and sudden cardiac death: results from the DISCOVERY trial and Oregon sudden unexpected death study. J Am Heart Assoc 5: e003905, 2016. doi:10.1161/JAHA.116.003905. 27895044 PMC5210425

[46] Brieger K, Schiavone S, Miller FJ Jr, Krause KH. Reactive oxygen species: from health to disease. Swiss Med Wkly 142: w13659, 2012. doi:10.4414/smw.2012.13659. 22903797

[47] Robinson BH. Human complex I deficiency: clinical spectrum and involvement of oxygen free radicals in the pathogenicity of the defect. Biochim Biophys Acta 1364: 271–286, 1998. doi:10.1016/s0005-2728(98)00033-4. 9593934

[48] Wisløff U, Najjar SM, Ellingsen O, Haram PM, Swoap S, Al-Share Q, Fernström M, Rezaei K, Lee SJ, Koch LG, Britton SL. Cardiovascular risk factors emerge after artificial selection for low aerobic capacity. Science 307: 418–420, 2005. doi:10.1126/science.1108177. 15662013

[49] Small GW, Rabins PV, Barry PP, Buckholtz NS, DeKosky ST, Ferris SH, Finkel SI, Gwyther LP, Khachaturian ZS, Lebowitz BD, McRae TD, Morris JC, Oakley F, Schneider LS, Streim JE, Sunderland T, Teri LA, Tune LE. Diagnosis and treatment of Alzheimer disease and related disorders. Consensus statement of the American Association for Geriatric Psychiatry, the Alzheimer's Association, and the American Geriatrics Society. JAMA 278: 1363–1371, 1997. 9343469

[50] Gianni D, Li A, Tesco G, McKay KM, Moore J, Raygor K, Rota M, Gwathmey JK, Dec GW, Aretz T, Leri A, Semigran MJ, Anversa P, Macgillivray TE, Tanzi RE, del Monte F. Protein aggregates and novel presenilin gene variants in idiopathic dilated cardiomyopathy. Circulation 121: 1216–1226, 2010. doi:10.1161/CIRCULATIONAHA.109.879510. 20194882 PMC2844798

[51] Gatt AP, Duncan OF, Attems J, Francis PT, Ballard CG, Bateman JM. Dementia in Parkinson's disease is associated with enhanced mitochondrial complex I deficiency. Mov Disord 31: 352–359, 2016. doi:10.1002/mds.26513. 26853899

